# Supplementary material for: Seminoma and Embryonal Carcinoma Footprints Identified by Analysis of Integrated Genome-Wide Epigenetic and Expression Profiles of Germ Cell Cancer Cell Lines
Source: PLoS One. 2014 Jun 2;9(6):e98330. doi: 10.1371/journal.pone.0098330 (PMC4041891; doi:10.1371/journal.pone.0098330)
Supplement: File S1 — ZIP file containing DMRforPairs output for significant regions. Please start from the html files. (ZIP) [file pone.0098330.s008.zip › figures/41036.pdf]

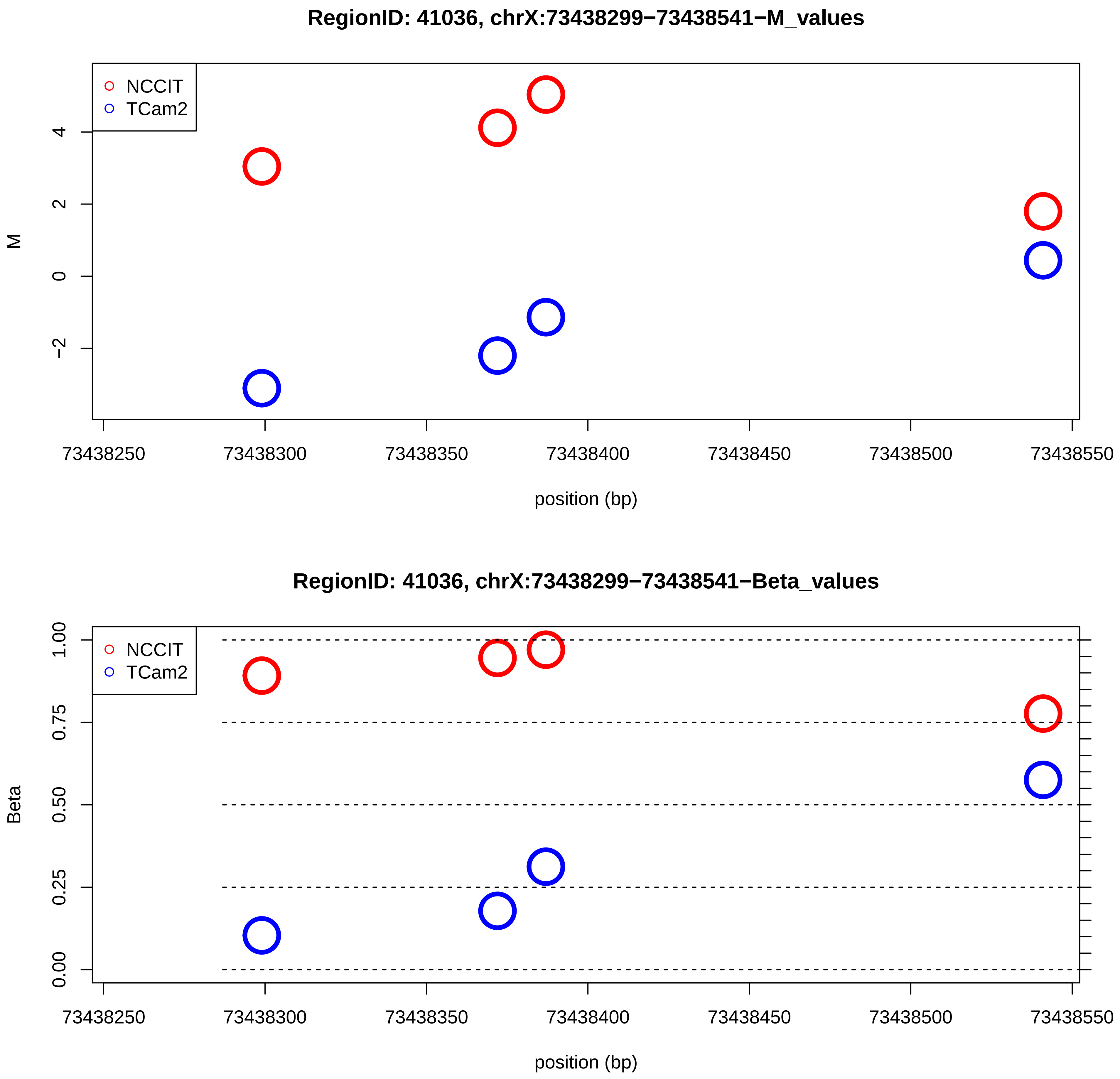
RegionID: 41036, chrX:73438299−73438541−M_values
NCCIT
TCam2
M
−2
0
2
4
73438250
73438300
73438350
73438400
73438450
73438500
73438550
position (bp)
RegionID: 41036, chrX:73438299−73438541−Beta_values
NCCIT
TCam2
Beta
0.00
0.25
0.50
0.75
1.00
73438250
73438300
73438350
73438400
73438450
73438500
73438550
position (bp)
